# Excess Cost of Telehealth Use in Radiation Oncology: A Medicare-Based Cohort Study

**DOI:** 10.7759/cureus.91978

**Published:** 2025-09-10

**Authors:** Kishan M Patel, Tarita Thomas, Dwight E Heron, Aaron Bush, Constantine A Mantz

**Affiliations:** 1 Radiation Oncology, Northwestern University Feinberg School of Medicine, Chicago, USA; 2 Radiation Oncology, Bon Secours Mercy Health System, Youngstown, USA; 3 Radiation Oncology, Costal Edge Radiation Oncology, Port Saint Lucie, USA; 4 Radiation Oncology, GenesisCare US, Fort Meyers, USA

**Keywords:** excess cost, hospitalizations, inpatient stays, medicare coding, patient care, risk mitigation, telehealth

## Abstract

Introduction: The COVID-19 pandemic necessitated rapid adaptations in healthcare delivery to ensure continuity of care while minimizing patient exposure to the virus. Telehealth emerged as a critical tool, supported by the Centers for Medicare and Medicaid Services (CMS) emergency waivers, which expanded telehealth services to include radiation treatment management. While telehealth has improved accessibility and convenience, its impact on clinical outcomes and healthcare utilization in oncology remains unclear. This study examines telehealth utilization during external beam radiation therapy episodes among Medicare beneficiaries, focusing on its association with emergency department (ED) visits, inpatient stays (IS), and related costs.

Methods: This retrospective cohort study analyzed Medicare beneficiaries who received external beam radiation therapy in outpatient hospital-based settings between 2020 and 2022. Radiation therapy episodes were constructed using the Medicare Standard Analytic Files (SAF) and included all treatment-related services provided over 90 days. Telehealth utilization was identified using Healthcare Common Procedure Coding System (HCPCS) codes, while ED visits and IS were determined from outpatient and inpatient claims. Multivariate logistic regression models were used to assess the association between telehealth use and healthcare utilization, adjusting for demographic and clinical risk factors. Costs were calculated based on Medicare payments and adjusted for inflation to account for changes in purchasing power over time.

Results: A total of 369,570 radiation therapy episodes were analyzed. Telehealth utilization declined from 9.6% in 2020 to 5.5% in 2022 but remained consistent within episodes where it was used. Patients utilizing telehealth had higher rates of ED visits (38.2% vs 30.2%) and IS (29.9% vs 21.6%) compared to those without telehealth. Multivariate analysis confirmed telehealth as a significant predictor of ED visits (p < 0.001, OR 1.057) and IS (p < 0.001, OR 1.080). Excess events attributed to telehealth use were estimated at 500 ED visits and 541 IS, with associated costs of $16.3 million during the study period.

Conclusion: Telehealth has become an essential component of cancer care, especially during the COVID-19 pandemic. Telehealth use during radiation therapy episodes was associated with increased healthcare utilization and costs, highlighting the need for optimized telehealth delivery models. Proactive care coordination and symptom management strategies may mitigate these risks. Further research is needed to explore the long-term impact of telehealth on patient outcomes and to refine its role in oncology care.

## Introduction

The COVID-19 pandemic, which was declared in March 2020, has profoundly impacted healthcare delivery, necessitating rapid adaptations to ensure continuity of care while minimizing patient exposure to healthcare environments. In response, the Centers for Medicare and Medicaid Services (CMS) implemented emergency declaration blanket waivers, which expanded telehealth services to include a broader range of care options, including radiation treatment management for cancer patients [1]. These policy changes enabled patients to access critical oncology services remotely, thereby reducing the need for face-to-face interactions with their physicians during a period of heightened concern for in-person contact. As a result, telehealth has been recognized for its ability to enhance accessibility and convenience, particularly during global emergencies such as the COVID-19 pandemic [2].

In oncology, telehealth has evolved to facilitate communication between interdisciplinary teams and patients, enable symptom management, and provide treatment guidance, all while reducing the burden of travel for vulnerable populations [3]. However, despite its apparent benefits, telehealth also presents challenges, including concerns about its impact on clinical outcomes, healthcare utilization, and the quality of patient-provider interactions.

This study examines the use and outcomes of telehealth in radiation oncology, focusing on its utilization during external beam radiation therapy episodes among Medicare beneficiaries. By analyzing data from 2020 to 2022, we aim to explore the association between telehealth use and healthcare utilization, namely emergency department visits and inpatient stays, while also considering the potential risks and limitations of telehealth in this context.

## Materials and methods

A retrospective cohort of Medicare beneficiaries who received external beam radiation therapy in hospital outpatient and provider-based departments between 2020 and 2022 was identified using the Medicare Standard Analytic Files. This study follows the Strengthening the Reporting of Observational Studies in Epidemiology (STROBE) reporting guideline [4].

Radiation therapy episode construction

Medicare 100% Standard Analytic Files (SAF) for calendar years 2000 through 2022 provided beneficiary-level claims and billing data for this study [5]. Radiation therapy episodes furnished in the facility setting (outpatient hospital-based) were constructed using Outpatient Revenue Center and Base Claims SAFs according to the method described by the RO-APM Final Rule and further detailed in a previous publication [6]. Each episode included all treatment delivery and related technical services provided to an individual patient over a 90-day period (global period). Episodes were excluded for any of the following conditions: (1) earliest treatment delivery service date occurring prior to March 31, 2020, the date the Centers for Medicare and Medicaid Services (CMS) issued an Interim Final Rule to include CPT 77427 (Radiation Treatment Management) on its temporary telehealth services list [7]; (2) episode starting after October 4, 2022, which would not permit a 90-day run-out period for episode completion; (3) no claims for external beam radiation therapy (EBRT) delivery identified during the episode; and (4) episodes for which payments were administered through Medicare Advantage health plans. The remaining episodes comprised the study cohort for this analysis.

Identification of telehealth, emergency department (ED), and inpatient stay (IS) related to radiation therapy episodes

In response to the COVID-19 Public Health Emergency (PHE) declaration in January 2020, CMS temporarily expanded existing telehealth provisions to include, among other services, weekly radiation treatment management performed and identified as CPT 77427 by radiation oncologists. To report a telehealth service and receive payment at the in-person rate, Medicare requires providers to conduct the service using an audio and video telecommunications system allowing for real-time interactive communication between providers and patients. In the context of a weekly visit with the radiation oncologist, the facility where the patient receives daily radiation therapy may serve as the originating site for a virtual visit and report Healthcare Common Procedure Coding System (HCPCS) code Q3014. This code is used to describe Medicare telehealth originating site fees and includes reimbursement for resources such as a location for the patient to receive the telehealth service [8].

Telehealth events were identified from Outpatient SAF claims by the reporting of the telehealth originating site facility fee HCPCS code Q3014 and abstracted. Those telehealth claims pertinent to individual radiation therapy episodes were then isolated and retained according to a two-step process of (1) extracting telehealth claims with beneficiary IDs matching those reported on episode claims and (2) extracting telehealth claims with dates of service occurring within the date range of treatment delivery services reported on episode claims on a same-beneficiary basis. Telehealth services provided by a radiation oncologist were further identified and isolated by the reporting of specific two-digit physician specialty codes on individual claims.

ED visits were identified from Outpatient and Inpatient SAF claims by the reporting of Revenue Center Codes 0450-0459 or 0981 and abstracted. These specific codes are used to identify emergency room (ER) services on medical claims, specifically for both inpatient and outpatient settings. Those ED visit claims relevant to radiation therapy episodes were isolated and retained using the same two-step process as described for telehealth claims with the following modifications: (1) ED claims with dates of service of up to and including 90 days after the last treatment delivery service for a given episode were also retained, and (2) multiple ED claims reporting the same date of service for the same beneficiary were deduplicated, with only one claim retained for that date of service. The first modification follows a previously used date range to assess post-acute care outcomes and costs among Medicare beneficiaries [9]. The second modification addresses the scenario of a patient transferring from one facility’s ED to another facility’s ED for inpatient admission, creating multiple same-day ED visit claims for a single event.

Inpatient stays were identified from Inpatient SAF claims, and those inpatient claims pertinent to radiation therapy episodes were isolated and retained using the same two-step process as described for telehealth claims, with a modification to allow for retention of inpatient claims with dates of admission of up to 90 days after the last treatment delivery service for a given episode. As with ED events, a 90-day post-acute period has been used to assess Medicare costs following an inpatient medical admission.

Determination of ED and inpatient stay Medicare costs

ED visit costs were calculated as the sum of Medicare payments reported on same-day claims identified within the Outpatient SAFs. Of note, payments for an ED visit resulting in admission are packaged into the associated diagnosis-related group payment under the Inpatient Prospective Payment System and therefore are not separately reimbursed. Costs related to inpatient stays (IS) were calculated as the sum of Medicare payments reported on Inpatient SAF claims. Costs related to physician services provided during ED visits and IS were calculated as the sum of Medicare payments reported on Carrier SAF claims. All costs were then adjusted for inflation using the Consumer Price Index for All Urban Consumers (CPI-U), with 2024 as the base year.

Assignment of risk attributes to episodes

As part of the development of the RO-APM, the Centers for Medicare and Medicaid Services (CMS) tested for and found certain clinical and demographic attributes to be predictive of the cost of care under the FFS payment system [10]. These attributes include age, sex, disability status, hierarchical condition category, and disease interactions [11]. These same attributes were identified for each beneficiary in this study within Medicare administrative data and then assigned to corresponding episodes.

Beneficiary age group, sex, and race information were abstracted from the Master Beneficiary Summary Files for calendar years 2020 through 2022. Cancer type was determined according to the plurality of International Classification of Diseases (ICD-10) diagnosis codes appearing on individual claims for each episode. Death within 30, 60, or 90 days after the start of an episode was determined by comparing the date of beneficiary death data contained within the Master Beneficiary Summary Files with the first date of service reported on individual episode claims. A major surgical procedure for a beneficiary either 90 days prior to or during an episode was determined by filtering Carrier, Outpatient, and Inpatient SAF claims against a Current Procedural Terminology (CPT) file of specified procedures developed by CMS for its RO-APM. Similarly, chemotherapy administration for a beneficiary was determined by filtering claims against a National Drug Code (NDC) file of specified cancer therapeutic agents.

In addition to the attributes determined by CMS, ED visits and inpatient stays within 90 days prior to an episode were also determined, as each has previously been reported to be a risk factor for increased costs and hospital readmissions within the Medicare system [12-14]. The method of identifying ED visits and inpatient stays described in the previous section was utilized, with the modification that any such events occurring within 90 days prior to an episode’s first date of service were identified and retained.

Statistical analyses

The assigned episode risk attributes, namely, age group, sex, race, cancer type, death within 30, 60, or 90 days after episode start, major surgical procedure, chemotherapy administration, and preceding ED visit or IS within 90 days before episode start, were each regressed against the outcome variables, ED visit and IS, using simple logistic regression. Generated coefficients were then tested for significance using the Wald test and a p-value of <0.05. All attributes were found to be significantly predictive of both outcome variables.

Separate multivariate logistic regression models were then constructed using these attributes as independent variables to test for their predictive significance for the occurrence of either an ED visit or IS. Regression coefficients were generated and tested using the Wald method, and a p-value of <0.05 was used for significance. Significant coefficients were then converted into odds ratios (OR) to calculate excess ED visits or IS related to the presence of the corresponding predictive variable.

All data preparation and statistical analyses were performed using KNIME Analytics Platform v.5.4.4 (KNIME, Zürich, Switzerland).

## Results

**Figure 1:**
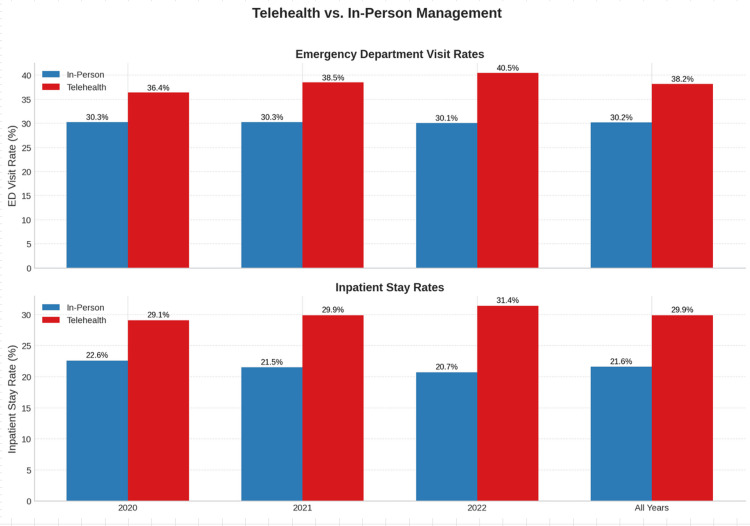
Bar graphs displaying annual percentage rates of emergency department visits (top) and inpatient stays (bottom) from 2020 to 2022, categorized by telehealth (red) versus in-person (blue) on-treatment visits. Telehealth use was associated with higher rates of both emergency department visits and inpatient stays across all years.

**Figure 2:**
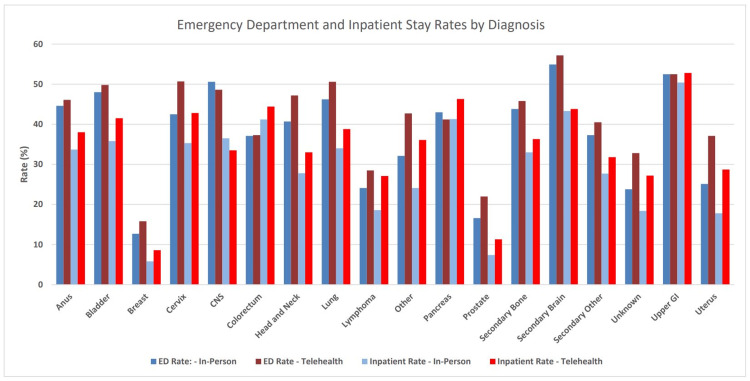
Bar graphs displaying percentage rates of emergency department visits and inpatient stays by anatomic diagnosis, categorized by telehealth (red) versus in-person (blue) on-treatment visits. Telehealth use was associated with higher rates of both emergency department visits and inpatient stays across all diagnoses except central nervous system and pancreatic malignancies.

A total of 369,570 episodes of external beam radiation therapy were delivered to Medicare beneficiaries in the outpatient hospital-based setting with dates of service beginning between March 31, 2020, and October 4, 2022. Telehealth utilization during radiation therapy episodes and corresponding rates of ED and IS over the study periods are illustrated in Figure 1. Telehealth utilization declined from 9.6% in 2020 to 5.5% in 2022 for all episodes. Within episodes where telehealth was used, utilization remained stable at one-half of all five-fraction (i.e., weekly) intervals throughout the study period. Compared to episodes without telehealth utilization and across all years, ED visits (38.2% vs 30.2%) and IS (29.9% vs 21.6%) during and 90 days post-episode were observed to be greater when telehealth was used. Distributions of telehealth utilization, ED visits, and IS according to cancer type are shown in Figure 2. Supplemental data are provided in the Appendices. 

After adjusting for the effects of all other independent variables, multivariate logistic regression identified telehealth utilization during the radiation therapy episode as significantly predictive of both outcome variables, ED visit (p <0.001, OR 1.057) and IS (p <0.001, OR 1.080). Using these odds ratios, the excess number of ED visits and IS related to telehealth use was estimated to be 500 and 541 events, respectively. Average Medicare costs for the entire study cohort were calculated as $1,265 per ED visit and $29,032 per IS on an inflation-adjusted basis, inclusive of all facility and provider Medicare payments. Therefore, estimated aggregate excess costs related to telehealth use were $632,486 for ED visits, $15,706,463 for IS, and $16,338,949 in total during the study period. Excess event and related cost data are presented in Table 1. 

**Table 1:** Excess ED and Inpatient utilization costs related to telehealth utilization OR: odds ratio, ED: emergency department, Inpt: inpatient.

	Episodes	ED Visits	Inpt Stays	OR, ED Visits	OR, Inpt Stays	Excess ED Events	Excess Inpt Events	Excess ED Costs	Excess Inpt Costs	Excess Total Costs
Telehealth	24,467	9,338	7,323	1.057	1.080	500	541	$632,486	$15,706,463	$16,338,949

Effects of telehealth services in patients receiving concurrent chemoradiotherapy

A subset analysis of beneficiaries who received concurrent chemotherapy during a radiation therapy episode was performed to evaluate the relative effects of telehealth services provided by either a radiation oncologist or a medical oncologist on ED or IS risk. This cohort was identified by the reporting of chemotherapy administration and supply codes among outpatient claims with dates of service falling within the service date ranges of radiation therapy episodes for the same beneficiaries. Each telehealth service was assigned to a radiation oncology or medical oncology category according to the physician specialty code indicated on the claim. A total of 59,716 episodes were identified with concurrent chemotherapy and radiation therapy delivery. The regression model was then analyzed for this cohort subset using the same input variables as previously applied to the entire cohort. Radiation oncology telehealth services were not significantly predictive of either ED visits or IS, whereas medical oncology telehealth services were significantly predictive of excess events for both (p <0.001 for both ED visits and inpatient stays). A second subset regression analysis was conducted on the remaining 309,854 episodes for which concurrent chemotherapy and radiation therapy were not performed. Telehealth services during radiation therapy episodes were assigned to a radiation oncology or any other specialty category. For this cohort, radiation oncology telehealth services were found to be significantly predictive of both excess ED and IS during the episode and the 90-day post-acute period (p <0.001 for both ED visits and IS).

## Discussion

Key findings

To our knowledge, this is the first analysis to identify patterns of telehealth use during radiation therapy episodes and its subsequent effects on emergency and inpatient service utilization in the Medicare population. While telehealth use overall declined from 9.6% of episodes in 2020 to 5.5% in 2022, its utilization remained consistent within treatment episodes when used. Telehealth visits accounted for roughly one-half of weekly treatment visits over the study period. Overall, patients receiving telehealth visits had statistically higher rates of emergency department use (38.2% vs 30.2%) and inpatient stays (29.9% vs 21.6%) than patients exclusively seen and examined by their physicians in person, corroborating earlier literature suggesting that while telehealth improves access, it could compromise other indicators of care quality, reinforcing that in-person encounters and examinations are critical in determining appropriate management decisions [15].

The statistical analysis revealed that telehealth use during a radiation therapy episode remained a significant independent predictor of excess emergency department visits and inpatient stays when adjusting for the effects of other clinical factors. This effect was observed regardless of disease site, for example, breast versus colon. Additional healthcare costs attributed to excess emergency and inpatient care related to telehealth use were estimated at $16.3 million during the study period. If telehealth were employed for all radiation therapy episodes over the same period, then the extrapolated excess costs would be estimated to approach $230 million.

The standard of care for the management of radiation oncology patients prior to the COVID-19 pandemic was weekly in-person physician visits to evaluate tolerability, address treatment-related side effects, and coordinate supportive care efforts. These data suggest that telehealth substitution may adversely affect the quality of care and increase costs to the healthcare system [6,16]. However, the data do not reflect patient satisfaction based on the quality of their care. Patient satisfaction was not compared in this review.

A subset analysis of patients receiving concurrent chemotherapy demonstrated notable differences in outcomes based on the specialty of the provider delivering telehealth care. Specifically, telehealth furnished by medical oncologists was significantly associated with increased ED visits and inpatient stays. In contrast, this effect was not observed when radiation oncologists provided telehealth for the same cohort. Several interpretations are possible. One explanation may relate to differences in clinical workflow or patient acuity: medical oncologists may be more likely to manage systemic side effects that require complex symptom triage or escalation of care, making remote assessment less effective. Alternatively, it is possible that these visits are markers of higher baseline risk or disease burden, which may not be fully adjusted for in claims data despite multivariable modeling. Conversely, the lack of association for radiation oncologist-furnished telehealth could reflect more protocol-driven care during radiation therapy, with better-established pathways for supportive care or in-person escalation when necessary.

However, the observed differences also raise questions about the consistency and quality of telehealth delivery across specialties. If valid, these findings suggest that the utility of telehealth may be highly context dependent and that specialty-specific training or infrastructure may be necessary to optimize outcomes. That said, this was a retrospective, observational analysis, and unmeasured variables, such as differences in visit content, patient communication preferences, or institutional protocols, may also confound outcomes. Further prospective investigation is warranted to clarify whether this differential effect reflects an actual difference in care quality or is an artifact of care setting and patient selection [4].

Implications for policy and practice

These findings have significant implications for policymakers and healthcare providers alike. While telehealth was a necessary resource during the COVID-19 pandemic, its subsequently increased healthcare utilization and related costs highlight the need to reconsider telehealth delivery models, particularly for patients with cancer. Innovative mitigation strategies employing proactive care coordination and symptom management need to be developed and integrated into telehealth services to reduce the risks of excess emergency visits and hospitalizations [14-17]. If telehealth is to remain a mechanism of care delivery, improvements in current practice are needed to mitigate adverse outcomes and costs in the post-pandemic period.

Limitations and future research

This study has several limitations. By the nature of the resources used, the study is retrospective, which subjects it to potential confounding factors that may not have been fully accounted for in the analysis. Additionally, telehealth utilization was identified using Medicare claims data, which may not capture all telehealth interactions or their qualitative aspects, such as patient satisfaction or clinical effectiveness. We were not able to assess the clinical impact on long-term treatment outcomes as a function of ED visits or IS. Furthermore, the study focused exclusively on Medicare beneficiaries, which limits the generalizability of the findings to other populations. Future research should explore the long-term outcomes of telehealth use in cancer-related care with an impact on quality of life, survival, and other patient-reported outcomes [4,6].

## Conclusions

Telehealth has significantly transformed the landscape of medicine and cancer care, particularly during the COVID-19 pandemic. It has made accessing treatment more convenient, allowing patients to receive portions of care from the comfort of their homes. However, our study highlights some significant and concerning challenges associated with the use of telehealth during radiation therapy. We found that it can lead to increased healthcare utilization and higher costs for patients. These insights underscore the need to refine and improve telehealth delivery models to enable enhanced patient outcomes while maintaining affordable care through additional targeted supportive care, such as potential symptom-management navigators, oncology triage, or fast-track emergency evaluation with associated payment modes. Moving forward, we must continue to invest in research and policy development to ensure a sustainable future. This will help unlock the full potential of telehealth in oncology while also addressing any limitations it may have. 

## Appendices

**Table 2:** Telehealth utilization by radiation oncologists during radiation therapy episodes and corresponding rates of emergency department (ED) and inpatient utilization during CY 2020-2022 TH: telehealth, ED: emergency department. TH visits per episode = average number of telehealth visits during a radiation therapy episode. TH visits per 5 fractions = average rate of TH use per week (i.e., every 5 fractions) during a radiation therapy episode; 0.5 indicates 50% of all weekly visits were performed using TH. Fractions = number of radiation treatments.

Telehealth	Episodes	Fractions	TH Visits	TH Visits per Episode	TH Visits per 5 Fractions	ED Visits	Inpatient Stays
No	345,103	-	-	-	-	104,365 (30.2%)	74,532 (21.6%)
Yes	24,467	423,982	44,604	1.8	0.5	9,338 (38.2%)	7,323 (29.9%)

**Table 3:** Telehealth utilization, ED visits, and inpatient stays according to cancer type ED: emergency department, Inpt = inpatient, GI: gastrointestinal, CNS: central nervous system.

		No Telehealth						Telehealth					
Diagnosis	Total Episodes	Episodes	% Total	ED Visits	Inpt Stays	ED Rate	Inpt Rate	Episodes	% Total	ED Visit	Inpt Stays	ED Rate	Inpt Rate
Anus	3,792	3,547	1.0%	1,583	1,194	0.446	0.337	245	1.0%	113	93	0.461	0.380
Bladder	5,374	4,972	1.4%	2,389	1,782	0.480	0.358	402	1.6%	200	167	0.498	0.415
Breast	78,109	74,370	21.6%	9,465	4,327	0.127	0.058	3,739	15.3%	591	321	0.158	0.086
Cervix	1,842	1,704	0.5%	724	602	0.425	0.353	138	0.6%	70	59	0.507	0.428
CNS	6,685	5,775	1.7%	2,920	2,106	0.506	0.365	910	3.7%	442	305	0.486	0.335
Colorectum	9,289	8,651	2.5%	3,213	3,568	0.371	0.412	638	2.6%	238	283	0.373	0.444
Head and neck	21,556	20,192	5.9%	8,226	5,610	0.407	0.278	1,364	5.6%	644	450	0.472	0.330
Lung	39,139	36,868	10.7%	17,018	12,521	0.462	0.340	2,271	9.3%	1,148	881	0.506	0.388
Lymphoma	12,224	11,334	3.3%	2,729	2,108	0.241	0.186	890	3.6%	254	241	0.285	0.271
Other	47,366	44,012	12.8%	14,143	10,618	0.321	0.241	3,354	13.7%	1,432	1,210	0.427	0.361
Pancreas	4,441	3,987	1.2%	1,715	1,647	0.430	0.413	454	1.9%	187	210	0.412	0.463
Prostate	60,001	57,452	16.6%	9,543	4,278	0.166	0.074	2,549	10.4%	560	289	0.220	0.113
Secondary bone	32,598	29,179	8.5%	12,771	9,631	0.438	0.330	3,419	14.0%	1,565	1,241	0.458	0.363
Secondary brain	11,286	10,319	3.0%	5,662	4,466	0.549	0.433	967	4.0%	553	424	0.572	0.438
Secondary other	15,872	14,134	4.1%	5,273	3,921	0.373	0.277	1,738	7.1%	704	553	0.405	0.318
Unknown	3,707	3,582	1.0%	852	660	0.238	0.184	125	0.5%	41	34	0.328	0.272
Upper GI	9,467	8,642	2.5%	4,540	4,356	0.525	0.504	825	3.4%	433	436	0.525	0.528
Uterus	6,822	6,383	1.8%	1,599	1,137	0.251	0.178	439	1.8%	163	126	0.371	0.287
Total	369,570	345,103	100%	104,365	74,532	0.302	0.216	24,467	100%	9,338	7,323	0.382	0.299
